# Record Hospital Services

**Published:** 1905-05-13

**Authors:** 


### RECORD HOSPITAL SERVICES.

### DR. O'REILLEY AND THE TORONTO GENERAL HOSPITAL.

Dr. Charles O'Reilley has enjoyed 40 years of hospital life in Canada. He took his degree at the Macgill University, and became medical superintendent at the Hamilton City Hospital in 1867. In January 1876 he was appointed medical superintendent at the General Hospital, Toronto, which post he will vacate at the end of 30 years' service. Under his management the General Hospital has made great strides, and is at the present time, with its 400 beds, the largest in the Dominion of Canada, and one of the most efficient in North America.

Under Dr. O'Reilley the General Hospital has been increased by the addition of the Andrew Mercer Eye and Ear Infirmary, the Pavilion for Women, the Burnside Maternity Hospital, and the erection of a large west wing for the reception of patients. In 1881 the Training School for Nurses was inaugurated, and it now has accommodation for 80 nurses, and the graduates of this school occupy nearly all the important positions in the schools and hospitals of Ontario. In connection with the General Hospital an Emergency Hospital has been opened in Toronto, where 2,500 patients are dealt with each year, the severer cases being forwarded to the General Hospital by ambulance.

Dr. O'Reilley has received the honorary medical degrees of the Trinity University, has been one of the examiners for the University of Toronto, and is at present clinical examiner for the latter and for the Ontario Medical Council.

Dr. O'Reilley is widely known as one of the most experienced medical superintendents in North America, and his long services are highly appreciated throughout the Dominion. The board of the General Hospital have granted Dr. O'Reilley a year's leave of absence on full pay, when he will retire from the service with a pension. There is a wide feeling in Canada that Dr. O'Reilley has well earned some special recognition, and we hope that the Governor-General may see his way to recommend Dr. O'Reilley for some mark of distinction when the Canadian honours are distributed on Federation Day, 1905.

